# Valorization of By-Products from Commercial Fish Species: Extraction and Chemical Properties of Skin Gelatins

**DOI:** 10.3390/molecules22091545

**Published:** 2017-09-14

**Authors:** Sérgio C. Sousa, José A. Vázquez, Ricardo I. Pérez-Martín, Ana P. Carvalho, Ana M. Gomes

**Affiliations:** 1 CBQF—Centro de Biotecnologia e Química Fina—Laboratório Associado, Escola Superior de Biotecnologia, Universidade Católica Portuguesa/Porto, Rua Arquiteto Lobão Vital, Apartado 2511, 4202-401 Porto, Portugal; sdcsousa2@gmail.com (S.C.S.); amgomes@porto.ucp.pt (A.M.G.); 2 Grupo de Reciclado y Valorización de Materiales Residuales (REVAL), Instituto de Investigacións Mariñas (IIM-CSIC) r/Eduardo Cabello, 6. Vigo-36208 Galicia, Spain; 3 Grupo de Bioquímica de Alimentos, Instituto de Investigacións Mariñas (IIM-CSIC) r/Eduardo Cabello, 6. Vigo-36208 Galicia, Spain; ricardo@iim.csic.es; 4 REQUIMTE/LAQV, Instituto Superior de Engenharia do Porto, Porto Polytechnic Institute, Rua Dr. António Bernardino de Almeida 431, 4294-015 Porto, Portugal

**Keywords:** extraction, fish gelatin, marine by-products, microstructural properties, rheological properties, textural properties

## Abstract

Fish skins constitute an important fraction of the enormous amount of wastes produced by the fish processing industry, part of which may be valorized through the extraction of gelatins. This research exploited the extraction and characterization of gelatins from the skin of three seawater fish species, namely yellowfin tuna (*Thunnus albacares*), blue shark (*Prionace glauca*), and greenland halibut (*Reinhardtius hippoglossoides*). Characterization included chemical composition, rheology, structure, texture, and molecular weight, whereas extraction studies intended to reduce costly steps during extraction process (reagents concentration, water consumption, and time of processing), while maintaining extraction efficiency. Chemical and physical characterization of the obtained gelatins revealed that the species from which the gelatin was extracted, as well as the heat treatment used, were key parameters in order to obtain a final product with specific properties. Therefore, the extraction conditions selected during gelatin production will drive its utilization into markets with well-defined specifications, where the necessity of unique products is being claimed. Such achievements are of utmost importance to the food industry, by paving the way to the introduction in the market of gelatins with distinct rheological and textural properties, which enables them to enlarge their range of applications.

## 1. Introduction

Gelatin is a soluble protein obtained by partial hydrolysis of collagen, constituting the main protein of connective tissue and the most abundant protein in mammals, birds, and fish [1,2]. The stabilization of collagen molecules is obtained by intra- and inter-chain hydrogen bonding, which results from an almost continuous repetition of the Gly-Pro-Hyp sequences. Covalent bonds also reinforce these molecules, constituting the basic unit of collagen fibrils [2]. The structure formed by many of these cross-linked collagen fibrils is responsible for the typically strong and rigid nature of skin, tendons, and bones. To be suitable for extraction, adequate swelling and solubilization of the insoluble native collagen is required. This is achieved through a chemical pre-treatment to disorganize the protein structure, which will break non-covalent bonds, usually by means of alkaline and acid solutions. The hydrogen and covalent bonds are afterwards cleaved by heat treatment in water, at temperatures higher than 40 °C, which results in a helix-to-coil transition and conversion into soluble gelatin [3]. The degree of collagen conversion into gelatin relates to the severity of the pre-treatment and the extraction process, as a function of pH, temperature, and extraction time. Depending on the pre-treatment (acid or alkaline), two types of gelatin are obtainable, namely type-A (isoelectric point at pH ~ 8–9) and type-B (isoelectric point at pH ~ 4–5), respectively.

Commercial gelatins are usually obtained from the skin and bones of cows and pigs. However, alternative sources are being required, due to the non-acceptance of the source of the raw materials by social or religious considerations [4], as well as the potential health risks such as the occurrence of bovine spongiform encephalopathy (BSE) [5]. Furthermore, the availability of gelatins from different sources and thus with different properties, expands their potential applications.

The fish processing industry is responsible for a large quantity of wastes, including skins, bones, fins, scales, and swim bladders, constituting 36% (and may represent up to 60%) of the raw material mass [4,6]. Since environmental laws restricting the disposal of fish discards are increasing, there is a strong demand towards the full utilization of aquatic resources. Fish gelatin is a relatively high molecular weight protein, which presents unique functional and technological properties [7]. In fish skins, reasonably mild acid treatment is enough to effect collagen solubilization, due to the acid liability of crosslinking in their immature collagens [3]. In recent years, various studies have focused on methodologies to obtain gelatins from fish wastes, as well as on the properties of such gelatins. In general, those studies are based on the combination of alkaline and mineral acid washes, followed by extraction at medium-high temperatures (40–90 °C). However, the duration of processing, water consumption and reagent concentrations are all high, and thus their minimization is needed [4,6,8,9,10,11,12].

Gelatin displays various functional roles in food processing and formulations, generally divided into two groups: those associated with the gelling process and those related to the surface behavior of the gelatin. Since none of the hydrocolloids currently on the market are capable of covering all of the abovementioned properties in all applications, different gelatin products with different characteristics may find a place in the market, depending on the major characteristics desired for a specific product. This situation may be a key advantage to the use of fish-based gelatins, as fish skins have a significant potential for the production of high-quality gelatin with widespread melting and gelling temperatures (in fact, over a much wider range than mammalian gelatins), yet still with sufficiently high gel strength and viscosity. Additionally, the lower melting point of cold-water fish based gelatin provides an enhancement of flavor release, fruit aroma, and melt rate in water gel desserts [13].

The present work studied the extraction process and physicochemical characterization of gelatins obtained from the skins of three commercially important seawater fish species in southern European countries (yellowfin tuna (YT)—*Thunnus albacares*, blue shark (BS)—*Prionace glauca*, and greenland halibut (GH)—*Reinhardtius hippoglossoides*), all extracted at two different temperatures (initially at 45 °C and subsequently at 80 °C), except for greenland halibut, which was only extracted at 45 °C.

## 2. Results and Discussion

### 2.1. Extraction Process and Chemical Composition

The extraction of water-soluble gelatins generally consists of a combination of several alkaline and acid washes, for protein breakdown and skin preparation to subsequent thermal extraction of hydrolyzed collagen [14,15,16]. The original extraction procedure, patented by Grossman and Bergman [14], describes three alkaline treatments (40 min/0.2% NaOH/1:14 (*w*/*v*)), three mineral acid washes (40 min/0.2% H_2_SO_4_/1:14 (*w*/*v*)) and three organic acid treatments (40 min/1% citric acid/1:14 (*w*/*v*)).

**Figure 1:**
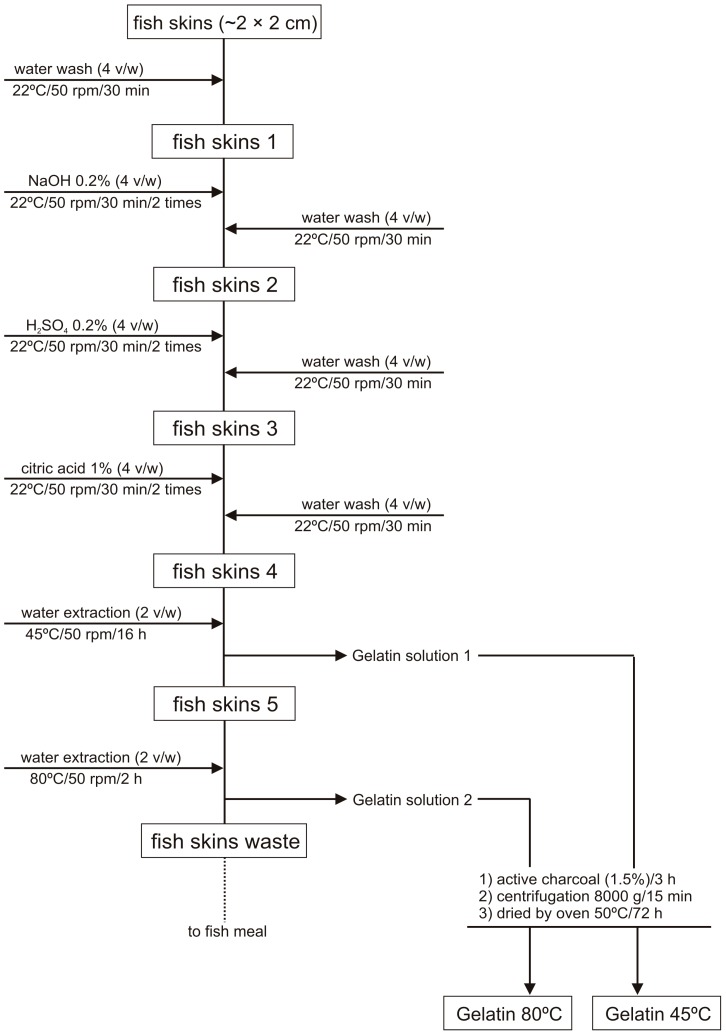
Flowchart of the steps performed for the optimized production of gelatins from fish skin by-products.

Figure 1 describes the set of sequential steps applied in the extraction of water-soluble gelatins from fish skin by-products. When compared with the abovementioned methods, our protocol used the same steps, although with ratios of only 1:4 (*w*/*v*) and fewer washes (two washes of 30 min for each stage). Additionally, the duration of washes in our protocol was shortened, when compared with the remaining protocols abovementioned, and water consumption and reagent concentrations were also reduced (optimization data unpublished). An additional step of cross-linking using citric acid was proposed previous to the water extraction step. Furthermore, room temperature conditions were established for all chemical washes, which enable a considerable energetic saving. Gudmundsson and Hafsteinsson [16] proposed three washes of 40 min for each treatment and a ratio of 1:7 (*w*/*v*), for cod skins. Limpisophon et al. [17] recovered gelatin from BS skins by applying three alkaline washes (30 min/0.2 M NaOH/10 °C) and one acid wash (3 h/0.05 M acetic acid) with ratios of 1:10 (*w*/*v*) in all treatments.

In conclusion, our modified procedure has advantages when compared with the conditions commonly reported in literature, which are especially time and water consuming, with moderate to high concentrations of alkali and acids.

One of the main challenges in the production of fish-based gelatin is to obtain a product with sufficient high gel strength and viscosity, yet with melting and gelling temperatures tailored to specific market demands. Such challenge may be achieved through the manipulation of conditions during the extraction process. In fact, the results from our study point out that the gelatin extracted from the same fish species but at different conditions (e.g., yellowfin tuna and blue shark skins treated at 45 °C and 80 °C) presents different physical and functional properties.

Results of skin chemical characterization are summarized in Table 1. The level of lipids presents in GH is remarkably high, compared to YT and BS. The yields of gelatin production for the different origins and temperatures of extraction were (as % *w*/*w* of fresh skin): 12.51 ± 1.27, 8.71 ± 0.91, 5.03 ± 0.34, 1.26 ± 0.09, and 1.15 ± 0.07 for YT-45 °C, BS-45 °C, GH-45 °C, YT-80 °C, and BS-80 °C, respectively. These values are in agreement with the content of total protein present in the skins (Table 1), with a higher concentration in YT, followed by BS and GH, respectively.

Values from extraction at 80 °C are significantly lower, as they correspond to a second extraction. Nevertheless, Sai-Ut and co-workers [18] obtained a 10.14% yield in the first extraction of gelatin from giant catfish, whereas the second extraction, at 60–90 °C yielded a recovery of 19.5%. However, experimental conditions and fish species were different, and therefore results are not directly comparable. The obtained values are higher than those obtained by Limpisophon et al. [17] for BS (3.93–5.2%), although similar with the findings of Jamilah et al. [19] (yields of 12.91% for red tilapia, 13.06% for walking catfish, and 11.7% for striped catfish), Eysturskarõ et al. [1] (yields of 8.9% for saithe skins), and Binsi et al. [8] (4.0% for bigeye snapper skin).

Proximate composition of gelatins is presented in Table 2. Between YT gelatins, extracted both at 45 and 80 °C, significant differences (*p* < 0.05) were only observed in humidity and protein content. Concerning BS gelatins, ash and fat were significantly different between extraction at 45 and 80 °C. When comparing gelatins from different species, although extracted at the same temperature, at 45 °C YT gelatin presented significant differences from BS in ash and fat, and from GH in moisture and ash; BS gelatin showed significant differences in every parameter when compared with GH, except for organic matter. When extraction was performed at 80 °C, YT and BS gelatins showed significant differences in moisture, ash, and protein. Gelatin moisture levels were relatively similar to the ones found for gelatin obtained from Nile tilapia skins (~8.5%), but higher than those found for gelatin extracted from the skins of giant catfish (~3.4%) [2]. Similar values to the ones found by those researchers were also found for YT gelatin ash contents. In any case, the water content is enough to limit the microbial contamination of the gelatins and suitable for their potential commercialization. However, fat (except for BS gelatin extracted at 45 °C) and protein contents were in all cases higher than the ones found in the previously mentioned work, for gelatins of both species tested (Nile tilapia and giant catfish). On the other hand, fishy odor and flavor was barely detected for the five types of gelatin produced.

### 2.2. Molecular Weight

**Figure 2:**
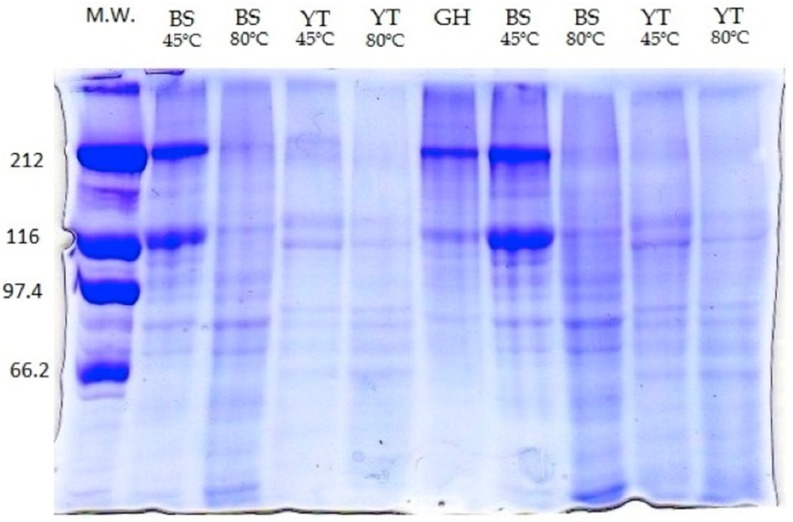
SDS-PAGE patterns of the protein marker (M.W.) and gelatins extracted from the different fish skins (YT: yellowfin tuna, BS: blue shark and GH: Greenland halibut), at different extraction temperature.

Molecular weight of the gelatins was determined from the results obtained in sodium dodecyl sulfate polyacrylamide gel electrophoresis (SDS-PAGE,) as presented in Figure 2.

Two major bands (212 and 116 kDa) were found in all gelatins, which represent the β and α-chain, respectively. YT gelatin showed one band at the molecular weight region of 212 kDa and two bands around the 116 kDa region, both with lower intensity for the 80 °C gelatin. Within the 97.4 and 66.2 kDa region four bands were present, with higher intensity in the 80 gelatin. BS gelatin extracted at 45 °C presented intense bands at 212 and 116 kDa. The 80 °C gelatin presented the same bands, however, with little intensity. For both gelatins, four bands were also observed within the 97.4 and 66.2 kDa region. These types of SDS profiles are in agreement with previously reported by giant catfish and tilapia [2] and Nile perch [20]. These authors have suggested that the appearance of low molecular weight components and the reduction of the intensity in the heaviest bands may be an indicator of partial collagen hydrolysis during gelatin manufacturing process, promoted by high extraction temperatures. GH gelatin mainly revealed the presence of the β and α-chain bands (212 and 116 kDa).

### 2.3. Amino Acid Composition

Amino acids compositions of the different gelatins showed that glycine (Gly) was the most abundant amino acid (~32%) but without significant differences (*p* > 0.05) between YT (324.0 ± 2.3 g·kg^−1^ at 45 °C, 320.2 ± 4.0 g·kg^−1^ at 80 °C) and BS (320.9 ± 1.7 g·kg^−1^ at 45 °C, 317.4 ± 6.2 g·kg^−1^ at 80 °C) gelatins, independently of extraction temperature. GH showed slightly higher glycine content (~33.6%). These values were higher than the ones found for gelatins obtained from other seawater fish skins as bigeye snapper [8], skates [21], but also freshwater fish skins such as red tilapia, walking catfish, and striped catfish [9], which all presented values between 20–21%. However, there are other studies where gelatins presented higher glycine percentage, like the case of 36.7% in grass carp skin gelatin [12]. Alanine (Ala) was the second most abundant amino acid, presenting values around 12% (116.4 ± 0.7 g·kg^−1^ for BS 45 °C and 123.0 ± 1.1g·kg^−1^ for YT 45 °C), except for GH (11%). These values are similar to the ones found for bigeye snapper (11.85%) [8]. The main limitations on the use of fish skin-originated gelatin rely on their sub-optimal physical and functional properties, when compared to mammalian-originated gelatin. The lower quality of some fish-based gelatins (e.g., lower gel strength, melting and setting points) is a result of their low content on the imino acids proline (Pro) and hidroxyproline (Hyp) [22]. Imino acids are important in the maintenance of the triple helical structure of the collagen, with Hyp believed to be an intervenient in the stabilization through its hydrogen bonding ability, conferred by the hydroxyl group. The content of these compounds varies significantly among fish species (as opposite to gelatin from mammalian sources—bovine and porcine—which presents similar characteristics), and is typically lower in cold-water fish species, when compared to warm-water fish species. Within the fish species studied, YT habitats are temperate/warm waters, as those from tropical Atlantic, whereas GH lives in cold waters (e.g., Atlantic and North Pacific) and BS appears everywhere (cold, temperate, and warm waters). Therefore, the present study allows the comparison of rheological properties of fish skin-originated gelatin from a large range of temperature habitats, and, in conformance with the aforementioned, results obtained show that GH presents the lower contents of Pro and Hyp. More specifically, and regarding Pro, YT gelatins presented the highest percentages (~11.2%, 111.9 ± 0.7 g·kg^−1^), followed by BS (~10.3%, 102.9 ± 0.5 g·kg^−1^) and GH (~9.3%, 93.2 ± 2.4 g·kg^−1^). Hydroxyproline content was similar between all gelatins, with a percentage around 7.5–8.5%. The imino acids content (Pro+Hyp) of GH (16.4%) was similar to grass carp gelatin, which was found to be 15.7% [12].

### 2.4. Rheology

**Figure 3:**
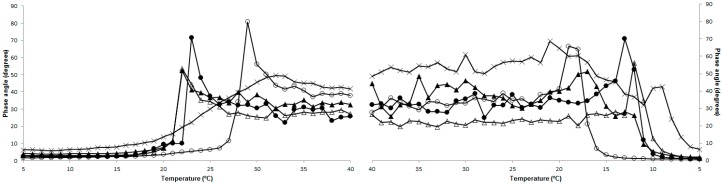
Phase angle evolution with increasing (**a**) and decreasing (**b**) temperature, of the different gelatins. Yellowfin tuna (YT) gelatin extracted at 45 °C (●), extracted at 45 °C and freeze-dried (■), and extracted at 80 °C (▲); BS gelatin extracted at 45 °C (○) and at 80 °C (∆); and GH gelatin extracted at 45 °C (x).

The dynamic viscoelastic parameters usually analyzed in rheology are phase angle, which measures how much the stress and strain are out of phase, the elasticity modulus (*G*’), which describes the amount of energy elastically stored, and the viscosity modulus (*G*’’), which gives an indication of the energy loss [12]. Our results, depicted in Figure 3, showed significant differences (*p* < 0.05) between the gelatins, according to the species from which the extraction was performed, but also according to the extraction temperature utilized in the process.

Melting and gelling points of the gelatins can be calculated from the sharp changes in phase angle observed throughout variation in temperature [23]. Results (Figure 3a) showed that melting temperature decreases with the increase of extraction temperature. YT and BS gelatins extracted at 80 °C presented the lower values (21.9 and 22 °C, respectively), BS gelatin extracted at 45 °C the highest (28.1 °C), and GH the second highest (25.3 °C). These values were similar to the ones obtained for gelatins from the skins of red tilapia (27.8 °C) and walking catfish (25 °C) [19]. Concerning gelling temperature (Figure 3b), a similar behavior was registered, with an increase in extraction temperature resulting in a decrease in gelling temperature. GH gelatin presented the lowest gelling temperature (11.2 °C), followed by BS gelatin extracted at 80 °C (11.7 °C). The highest gelling temperature was observed for BS gelatin extracted at 45 °C, with 19.5 °C.

**Figure 4:**
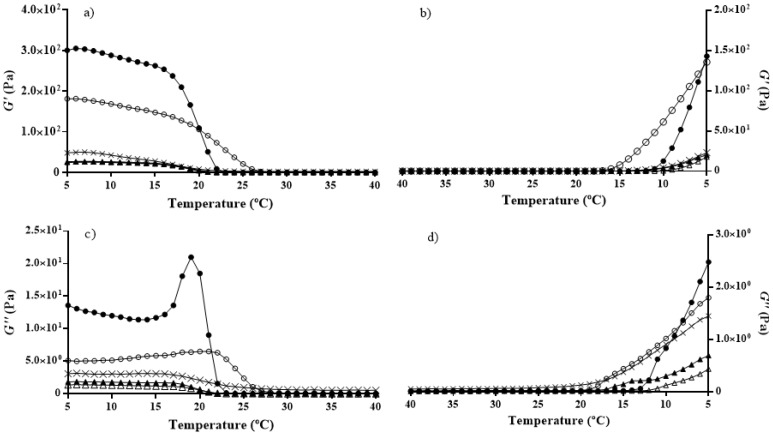
Elastic modulus (*G*’) (**a**,**b**) and viscosity modulus (*G*’’) (**c**,**d**) evolution with increasing (**a**,**c**) and decreasing (**b**,**d**) temperature, of the different gelatins. Yellowfin tuna (YT) gelatin extracted at 45 °C (●), extracted at 45 °C and freeze-dried (■), and extracted at 80 °C (▲); BS gelatin extracted at 45 °C (○) and at 80 °C (∆); and GH gelatin extracted at 45 °C (x).

Regarding the elasticity modulus (*G*’) and viscosity modulus (*G*’’) (Figure 4), YT gelatin extracted at 45 °C showed the highest values.

Results also showed gelatins extracted at lower temperature (45 °C) to possess the best viscoelastic properties. When temperature increased (Figure 4a,c), their elasticity and viscosity started to decrease later than the gelatins extracted at 80 °C, and when temperature decreased (Figure 4b,d), their values started to increase earlier (except for *G*’’ of YT gelatin at 45 °C vs. 80 °C). These results regarding viscoelastic properties can be a consequence of the gelatins molecular weights, since α-chains present a higher ability to refold [12] and, as previously mentioned, extraction at 80 °C produced gelatins with lower content of these chains, when compared to extraction at 45 °C.

### 2.5. Textural Analysis

Textural analysis results (Table 3) showed BS gelatin, extracted at 45 °C, to be the gelatin with the highest bloom (189). Intermediate Bloom of 107 and 97 was presented by YT extracted at both 45 °C and 80 °C, respectively, and GH presented the lowest Bloom (14). These values were somewhat similar to the ones obtained in other studies for walking and striped catfish skins (239 and 147, respectively) and bigeye snapper (108) [8,9]. These values are, however, lower than the ones found for grass carp (267) [24], and the Bloom value of YT extracted at 45 °C (107), is much smaller than the one obtained by Cho et al. (426) [25].

Bloom value is one of the main parameters studied in textural analysis, when gelatins are concerned, as it correspond to the gel strength. Gel strength is a function of the interactions, which are determined by the amino acids composition, namely, the α- and β-components. The gelatin strength increases with increasing amounts of these components [10]. These results are, therefore, in agreement with the molecular weight results, where it was observed that higher extraction temperatures degraded the proteins, which impacted on the gel strength, as it can be observed in the Bloom values.

Concerning the other parameters measured, as expected, there was a similar behavior to that obtained for gel strength (Bloom). Rupture strength measures the force that has to be applied for the gelatin to rupture, and was the highest in BS gelatin, extracted at 45 °C, indicating a stronger gelatin. YT presented intermediate values, with no significant difference (*p* > 0.05) between the two extraction temperatures, and GH presented the lowest value. Adhesiveness was similar among the gelatins, except for GH, which presented a considerably lower adhesiveness. Brittleness is the tendency of a material to fracture or fail, and the results concerning this parameter showed BS, extracted at 45 °C, to have the highest value, which means that this gelatin had a higher tendency to break, without deforming, which was accepted, since it presented the highest Bloom. YT gelatins presented similar lower values, independent of the extraction temperature, and GH gelatin was, once again, the gelatin with the lowest value.

### 2.6. Structure

**Figure 5:**
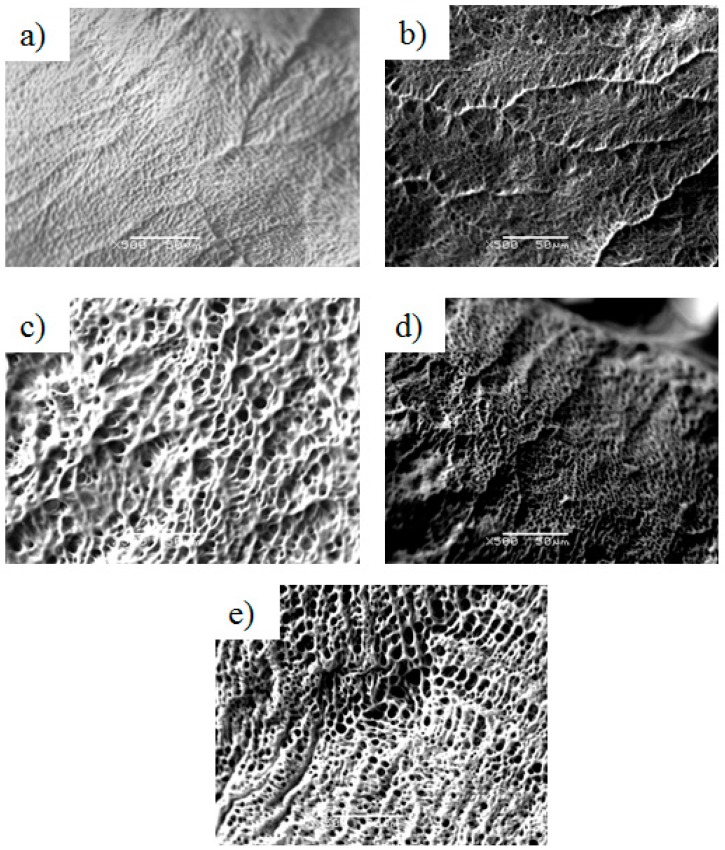
Scanning electron microscope (SEM) micrographs of the different gelatins. BS 45 °C (**a**); BS 80 °C (**b**); YT 45 °C (**c**); YT 80 °C (**d**); and GH 45 °C (**e**). The marker in the micrographs corresponds to 50 μm.

Gelatin structure was analyzed by SEM and results are shown in Figure 5. SEM images showed the structure of YT gelatins (Figure 5c,d) to be similar, independent of extraction temperature, which is consistent with the previous findings concerning these gelatins integrity and strength. BS gelatins (Figure 5a,b) showed significant differences, with gelatin extracted at 80 °C (Figure 5b) revealing a more porous network. In the BS gelatin extracted at 45 °C (Figure 5a), the network appears to only present depressions and not pores. This type of porosity (almost inexistent) can explain the increased “hardness” of this gelatin, when compared with the other gelatins. GH gelatin (Figure 5e) was the gelatin that presented the network with more and larger pores, which can justify the results of the previous analysis, where this gelatin showed to have the least integrity among the tested gelatins.

## 3. Materials and Methods

### 3.1. Samples

Skin fish wastes were kindly provided by Frinsa del Noroeste S.A. (YT—yellowfin tuna, *Thunnus albacares*), Propegal S.L. (BS—blue shark, *Prionace glauca*) and Fandicosta S.A. (GH—Greenland halibut, *Reinhardtius hippoglossoides*), being washed and stored at −20 °C until use. Prior to extraction (and still frozen), skins were cut in portions of ca. 2 × 2 cm.

### 3.2. Gelatin Extraction

The skin gelatins were extracted based on a modified method of Grossman and Bergman (1992) [14]. The preparation of all skin portions, previous to thermal extraction, was performed by a repetitive combination of alkaline and acid washes (Figure 1) in 1:4 (*w*/*v*) proportion. 

Water extraction of gelatins 1:2 (*w*/*v*) was carried out at 45 °C/16 h for YT, BS and GH treated skins, and a subsequent extraction of the same skins was performed at 80 °C/2 h (only for YT and BS). The obtained gelatin solutions were mixed with active charcoal (1.5%) for 3 h, for odor adsorption, and centrifuged at 8000× *g*/15 min. Finally, cleaned and deodorized gelatin solutions were oven-dried (50 °C/72 h).

Gelatin samples were prepared by dissolving 6.67% (*w*/*v*) gelatin in deionized water at room temperature for 30 min, followed by 30 min in a water bath at 60 °C (with constant agitation). Gelatin solutions were then left to cool in a refrigerator at 4 °C, for 16–18 h, prior to analysis [26].

### 3.3. Molecular Weight

Molecular weight of fish gelatines was determined by SDS-PAGE according to the method firstly described by Laemmli (1970) [27]. Protein solutions of 1 mg/mL were mixed at 1:1 (*v*/*v*) ratio with the sample buffer containing (*v*/*v*): 21.1% SDS 10%, 10.5% glycerol, 13.2% Tris–HCl pH 6.8 (0.5 M), 2.6% bromophenol blue 1%, and 6.32 mg/mL of dithiothreitol (DTT). Subsequently, they were heated for 5 min at 100 °C. About 15 μL of each sample was loaded onto the gels, made of 5% stacking and 7% separating gels, and subjected to electrophoresis at constant current of 35 mA per gel, using a Bio-Rad PowerPac 2000 Electrophoresis Power (Bio-Rad Laboratories, Hercules, CA, USA). The standard protein markers used to estimate molecular weights were myosin (212 kDa), β-galactosidase (116.0 kDa), phosphorylase b (97.4 kDa), and bovine serum albumin (66.2 kDa) (Protein MW Marker for SDS electrophoresis, Amresco LLC, Solon, OH, USA). After electrophoresis, protein was detected by staining with 0.1% Coomassie Brilliant Blue R (PhastGelR Blue R, Sigma-Aldrich, St. Louis, MO, USA), and distaining with 25% ethanol and 8% acetic acid solution.

### 3.4. Chemical Composition

Amino acid profiles of the gelatins were analyzed by ninhydrin reaction, using an amino acid analyzer (Biochrom 30 series, Biochrom Ltd., Cambridge, UK), according to the method of Moore et al. (1958) [28]. The moisture content was quantified by drying the gelatin solutions in an oven at 105 °C until a constant weight was obtained. Ash content was measured in an oven at 550 °C for 24 h. Crude protein was calculated by converting the total nitrogen concentration (6.11 × N) determined with the Kjeldahl [29]. This value of K-factor (6.11) was calculated by dividing the total content of amino acids (sum of amino acids weights per 100 g of gelatin) between the total nitrogen concentrations. Fat was determined as described in AOAC methods (Official Method 991.36) [30], using the Soxhlet system. Organic matter was calculated by difference between total weight of sample and moisture and ash content.

### 3.5. Rheology

Rheological analysis was performed on a Gemini Advanced Rheometer (Bohlin Instruments, Bristol, UK), coupled with a peltier unit. The viscoelastic properties of the gelatines were measured during heating and cooling from 5 to 40 °C and from 40 to 5 °C (with a 10 min interval between the ramps), respectively, with a heating/cooling ramp of 1 °C/min and a frequency of 1 Hz.

### 3.6. Texture

The texture profiles were analyzed in a TA.XT*plus* Texture Analyzer (Stable Micro Systems, Godalming, UK) with a 5 g load cell, equipped with a 0.5 diameter Teflon probe. A trigger force of 4 g and a penetration speed of 1 mm/s were utilized, with a penetration depth of 4 mm. Gel strength (expressed in g), rupture strength (expressed in g), adhesiveness (expressed in g) and brittleness (expressed in kg/s) were measured.

### 3.7. Structure

Gelatin structure was analyzed with a scanning electron microscope (SEM; JSM-5600LV, JEOL, Tokyo, Japan), coupled with a cold stage by peltier effect unit. Small cubes of gelatin samples were cut and placed in an observation stub, covered with double sided adhesive carbon tape (NEM, Nisshin, Japan), and immersed in liquid nitrogen. Immediately afterwards, the frozen sample was inserted in the SEM observation chamber, containing the cold stage unit, so that the sample would not defreeze. Observations were performed at a working distance of 8 mm, with an acceleration voltage of 15 kV and a spot-size of 36.

### 3.8. Statistical Analysis

Experiments, and analyses within each experiment, were performed in duplicate. Statistical analysis was performed with IBM SPSS Statistics for Windows, Version 20.0 software (IBM Corp, Armonk, NY, USA), and *t*-tests were performed for pairwise comparisons with 95% confidence.

## 4. Conclusions

Gelatins from skins of several seawater fish were extracted by a modified Grossman and Bergman method, in which the number and the duration of washes, as well as the water consumption, were largely reduced. Concerning the characterization of such gelatins, it was observed that the fish species from which they arise and the temperature play a key influence in their final properties. Higher extraction temperatures (80 °C) originated gelatins with lower values concerning rheological and textural parameters, lower molecular weight peptides and, consequently, lower structural integrity, probably as a consequence of protein chain degradation. Regarding fish species effect, YT presented “softer” gelatins than the ones obtained from BS skins. For almost every parameter tested, GH gelatin presented the lower values, which defined it as a “soft” gelatin, with very little physical integrity. The combination of extraction conditions and fish species allowed for the production of gelatins with unique characteristics, potentially applicable to very different market needs. Furthermore, there are other inherent advantages on using fish-based gelatins, such as the religious acceptability (they are acceptable for Islam and Judaism, the later with minimum restrictions), absence of risk related with BSE outbreaks, and diminution of by-products from fish processing industry, thus reducing amount of wastes generated.

## Figures and Tables

**Table 1:** Proximate composition of fish skins (g·kg^−1^). BS: blue shark, YT: yellowfin tuna and GH: greenland halibut.

Species	M	Ash	Fat	Pr-Nt
BS	760.3 ± 8.3	42.4 ± 2.4	2.4 ± 0.3	227.9 ± 9.7
YT	625.7 ± 24.0	6.7 ± 1.4	32.2 ± 7.2	323.8 ± 20.4
GH	554.4 ± 9.8	176.3 ± 12.1	106.2 ± 9.8	159.5 ± 4.2

Note: Values are average ± confidence interval (α = 0.05); M: moisture; Pr-Nt: protein as total nitrogen × 6.11.

**Table 2:** Proximate composition of fish gelatins (g·kg^−1^).

Gelatins	M	OM	Ash	Fat	Pr-Nt
BS 45 °C	96.7 ± 2.0	886.6 ± 9.2	16.7 ± 1.8	8.3 ± 0.6	929.0 ± 13.1
BS 80 °C	97.1 ± 0.9	897.9 ± 10.0	5.0 ± 0.4	26.6 ± 2.7	935.0 ± 10.8
YT 45 °C	99.7 ± 0.8	899.2 ± 6.7	1.1 ± 0.2	28.6 ± 2.9	906.0 ± 11.3
YT 80 °C	74.0 ± 2.1	924.3 ± 19.6	1.7 ± 0.7	25.5 ± 3.9	866.0 ± 16.3
GH	89.6 ± 2.1	901.1 ± 9.8	9.3 ± 1.2	29.3 ± 2.8	869.0 ± 14.4

Note: Values are average ± confidence interval (α = 0.05); M: moisture; OM: organic matter; Pr-Nt: protein as total nitrogen × 6.11.

**Table 3:** Textural analysis of the different gelatins.

Gelatins	Gel Strength (g)	Rupture Strength (g)	Adhesiveness (g)	Brittleness (kg·s^−1^)
BS 45 °C	189 ± 1 ^a^	653 ± 11 ^a^	−39 ± 12 ^a^	6.7 ± 0.2 ^a^
BS 80 °C	64 ± 1 ^b^	251 ± 14 ^b^	−37 ± 1 ^a^	2.1 ± 0.2 ^b^
YT 45 °C	107 ± 10 ^c^	380 ± 34 ^c^	−38 ± 5 ^a^	3.6 ± 0.1 ^c^
YT 80 °C	97 ± 13 ^c^	342 ± 72 ^b,c^	−43 ± 7 ^a^	3.0 ± 0.7 ^b,c^
GH 45 °C	14 ± 0 ^d^	46 ± 5 ^d^	−2 ± 2 ^b^	0.6 ± 0.1 ^d^

Note: Numerical values are average ± confidence interval (α = 0.05); a–d: values with the same superscript letter are not significantly different (*p* > 0.05).
